# Managing a Rare Case of Methicillin-Resistant Staphylococcus aureus (MRSA) Holocord With Intravenous Antibiotics

**DOI:** 10.7759/cureus.79918

**Published:** 2025-03-02

**Authors:** Kevin T Dao, Matthew X Perera, Sabrina A Yip, Kasey Fox

**Affiliations:** 1 Internal Medicine, UCLA Kern Medical, Bakersfield, USA

**Keywords:** broad spectrum antibiotics, holocord spinal epidural abscesses (hsea), medical management, methicillin resistant staphylococcus aureus, spinal epidural abscesses (sea), spinal subdural abscesses (ssa)

## Abstract

Holocord pathologies are diseases that include the entire spinal cord, and, in most instances, neurological cancers are the most common cause of holocord pathologies. However, in some rare instances, there are cases in which bacterial infections can extend into deeper spaces, causing spinal epidural abscesses (SEA), holocord SEA (HSEA), or even rarer spinal subdural abscesses (SSA). Current discussions surrounding the management of HSEA, SEA, or SSA primarily involve early surgical intervention and subsequent antibiotics. However, in this case, we present a patient with methicillin-resistant Staphylococcus aureus (MRSA) holocord subdural abscess, along with epidural and paraspinal abscesses who was treated with intravenous antibiotics and no surgical intervention. A discussion regarding this rare disease, along with the treatment of MRSA holocord, is also included.

## Introduction

Staphylococcus aureus is a Gram-positive diplococci bacterium commonly associated with skin and soft tissue infections and is considered one of the leading causes of worldwide bacterial infections. [1] In many instances, this microorganism, particularly its methicillin-resistant form, has been a leading cause of increasing mortality and morbidity with a diverse form of pathologies [2]. Abscess formation often occurs with these infections and can form in deeper tissues, including the spinal epidural space. However, spinal epidural abscesses (SEA) are relatively uncommon, with an incidence of 0.051% per admission, and S. aureus being the leading bacterial cause, with 25% of the proportion being methicillin-resistant S. aureus (MRSA) isolates [3,4]. Spinal subdural abscesses (SSA) and holocord SEA (HSEA) are even rarer entities - with fewer than 10 reported cases of HSEA as of 2019 - and could result in potentially catastrophic neurological consequences if not diagnosed and treated in a timely manner [4]. However, despite this disease being rare, certain co-morbidities have been associated with the development of HSEAs, such as intravenous drug use, alcohol use, immunocompromised states, and/or diabetes [5]. The current proposed management for HSEA and SSA seems to be primarily surgical intervention with IV antibiotics [4-6]. In fact, two studies noted that early detection and immediate surgical intervention, followed by antibiotics, lead to a good prognosis [7,8]. Given the rare occurrence of both HSEA and SSA, we would like to present a rare case of a 56-year-old male who developed both HSEA and SSA, which were managed with just IV antibiotics and supportive care.

## Case presentation

A 56-year-old male, with a medical history of intravenous methamphetamine use and hypertension, presented to the emergency department with complaints of generalized body aches, nausea, non-bilious/non-bloody vomiting, and non-bloody diarrhea. He had also noted a two-day history of progressively worsening neck and right-sided back pain localized to the cervical and lumbar regions, which he described as intermittent and non-radiating 10 out of 10 pain. The patient otherwise denied any associated numbness, tingling, paresthesia, bowel/bladder incontinence, or any other symptoms. On physical examination, he was febrile at a temperature of 38.3°C, tachypneic with a respiratory rate of 23 breaths per minute, and tachycardic with a heart rate of 110 beats per minute. The patient was also noted to be in moderate distress with increasing agitation. Brudzinski and Kernig's signs were positive. No peripheral edema, overt erythema, or swelling of the dermis were noted, and no track marks of bilateral upper and lower extremities were identified.

Initial laboratory evaluation revealed a markedly elevated white blood cell count (WBC) of 27.2 × 10^9^/L with neutrophilic predominance. Inflammatory markers were significantly elevated, including a C-reactive protein (CRP) level of 26.2 mg/L and an erythrocyte sedimentation rate (ESR) of 62 mm/hour (Table 1). Blood cultures were also done with immediate initiation of vancomycin, cefepime, ampicillin, and dexamethasone shortly after. A lumbar puncture was done, which revealed a purulent and turbid fluid. Further analysis of the cerebral spinal fluid (CSF) was done (Table 2) with a Gram stain of the CSF fluid pending.

**Table 1:** Initial blood work mmol/L = millimols/liter; mg/dL = milligrams/deciliter; units/L = units/liter; mg/dL = milligrams/deciliter; mm/hr = millimeters/hour; g/dL = grams/deciliter; fL = femtoliters; /mcL = cell/microliter

Lab Value	Value	Reference
Sodium	134 mmol/L	136 mmol/L–145 mmol/L
Potassium	3.6 mmol/L	3.5 mmol/L–5.1 mmol/L
Chloride	102 mmol/L	98 mmol/L–107 mmol/L
Calcium	8.5 mg/dL	8.5 mg/dL–10.1 mg/dL
Magnesium	1.8 mg/dL	1.8 mg/dL–2.4 mg/dL
Phosphorus	3.3 mg/dL	2.5 mg/dL–4.9 mg/dL
Alanine transaminase	44 units/L	13 units/L–61 units/L
Aspartate transaminase	22 units/L	15 units/L–37 units/L
Direct bilirubin	1.6 mg/dL	0 mg/dL–0.2 mg/dL
Total bilirubin	2.4 mg/dL	0 mg/dL–1 mg/dL
Erythrocyte sedimentation rate	62 mm/hr	<20 mm/hr
C-reactive protein (CRP)	26.20 mg/dL	<0.3 mg/dL
White blood count (WBC)	27.2×10^3/mcL	4.5×10^3^/mcL - 11×10^3^/mcL
Hemoglobin (Hgb)	14.1 g/dL	13.2 g/dL–17.4 g/dL
Mean corpuscular volume (MCV)	88.9 fL	80 fL–98 fL
Platelet	299×10^3^/mcL	150x10^3^/mcL–450x10^3^/mcL
Neutrophil %	91.3%	50%–75%
Lymphocyte %	1.9%	20%–45%
Bands %	11%	<12%
Monocyte %	3%	2%–12%
Eosinophil %	0%	<6%
Absolute neutrophil	29.7×10^3^/mcL	1.8×10^3^/mcL–7.7×10^3^/mcL
Absolute lymphocyte	0×10^3^/mcL	1.2×10^3^/mcL–4.5×10^3^/mcL
Absolute monocyte	0.9×10^3^/mcL	0.1×10^3/mcL–1×10^3^/mcL
Absolute eosinophil	0×10^3^/mcL	<0.7×10^3^/mcL

**Table 2:** Cerebral spinal fluid (CSF) analysis /mcL = cell/microliter; mg/dL = milligrams/deciliter; n/a = not available; WBC: white blood cell count

Lab Value	Value	Reference
CSF WBC	18,000/mcl	< 5
CSF neutrophil %	94%	n/a
CSF lymphocyte %	4%	n/a
CSF monocyte %	1%	n/a
CSF macrophage %	1%	n/a
CSF glucose level	7 mg/dL	40–75 mg/dL
CSF protein level	585 mg/dL	15–45 mg/dL

A computerized tomography (CT) scan of the brain and head with and without contrast was notable for mild atrophy, calcifications in the pineal region, and normal intracranial anatomy, without any acute intracranial pathology. CT of the cervical, thoracic, and lumbar spine showed various collections throughout the spinal cord along with multilevel degenerative disc disease (DDD) and severe bilateral foraminal narrowing.

**Figure 1:**
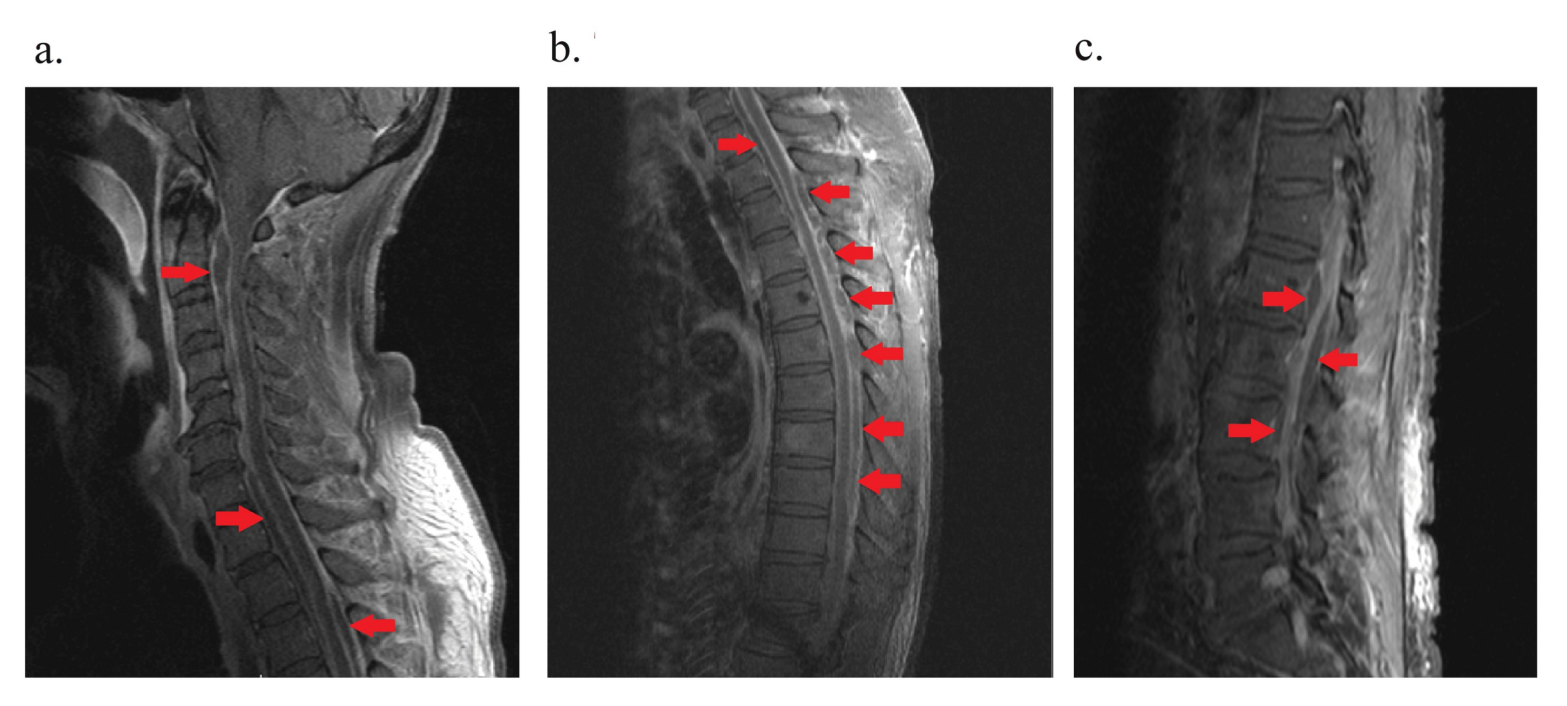
MRI of the cervical, thoracic, and lumbar spine saggital view 1a: Cervical spine; 1b: Thoracic spine; 1c. Lumbar spine The red arrows noted show various collections throughout the patient's spinal cord.

**Figure 2:**
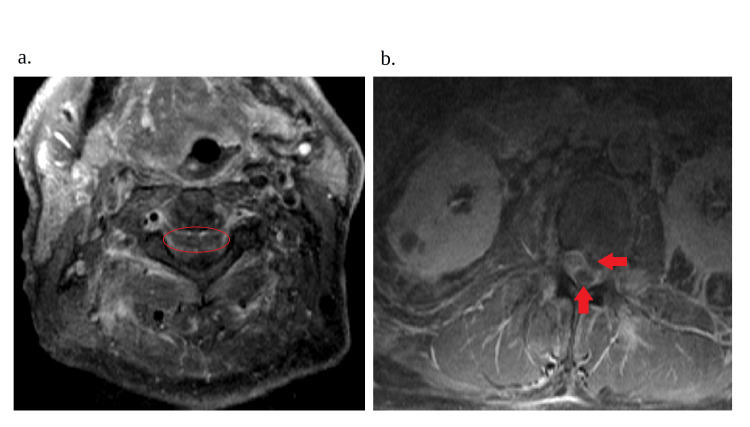
MRI of the spine transverse view 2a. Cervical spine and the red circle indicating a collection in the cervical region of the patient’s spinal cord.2b. Lumbar spine and red arrows showing a collection in the lumbar spine that wraps around the spinal cord.

Blood cultures and CSF cultures confirmed methicillin-resistant S. aureus (MRSA). The patient’s antibiotic regimen was adjusted to ampicillin 2 g every four hours and cefepime 2 g every eight hours. Vancomycin was changed to daptomycin 850 mg daily due to the patient developing acute kidney injury. The patient's mental status rapidly declined with agitation, and due to concerns about airway protection, the patient was intubated and upgraded to the intensive critical care unit. An MRI of the cervical, thoracic, and lumbar spine showed a large anterior epidural collection from the dens to C7 suggestive of an epidural abscess causing significant mass effect on the spinal cord, particularly at the C2 and C5-C6 levels (Figure 1a). The study also revealed multilevel DDD and severe bilateral foraminal narrowing. In addition, there were multiple epidural abscesses, including ventral abscesses at the C1, T2, and L1 levels (Figures 1a-2b), as well as posterior abscesses at the L5-S1 level (Figures 1c, 2b). The imaging also demonstrated intramuscular abscesses in the bilateral psoas muscles and micro-abscesses within the paraspinal muscles, consistent with myositis. These findings confirmed the diagnosis of extensive holocord subdural abscess with extensive epidural and paraspinal abscesses, complicated by severe myositis with diffuse leptomeningeal enhancement seen throughout the spinal cord.

Due to the diffuse process of the infection involving the entire spine, both intra- and extra-durally, our team began the process of transferring the patient to a higher level of care facility for neurosurgery intervention, which could not be performed at our facility, while continuing antibiotics and all other medical management while searching for an accepting facility. Cardiac evaluation via a transthoracic echocardiogram was noted to be unremarkable except for a left ventricular ejection fraction (LVEF) of 30-35%, but no evidence of mass or vegetations was appreciated. A transesophageal echocardiogram (TEE) was performed, which showed similar results.

Once repeated cultures showed only MRSA growth, ampicillin and cefepime were discontinued, and ceftaroline 600 mg IV every eight hours was added along with daptomycin 850 mg daily. After a few days in the ICU, the patient was able to slowly improve on the antibiotics and was eventually extubated with improving mentation. The patient was then downgraded to the medicine floor with a significant improvement in symptoms. At that time, the patient refused further efforts for transfer to a higher level of care and further imaging. Once repeat blood cultures were negative, the patient was discharged to acute rehabilitation with IV antibiotics, daptomycin and ceftaroline, for a total of six weeks with occupational and physical therapy with appropriate follow-up.

## Discussion

HSEA and SSA are rare infections, with an estimated incidence of 0.051% of hospital admissions, and can lead to severe rapid neurological deterioration [3,9,10]. Such widespread infections are generally unusual and underscores the aggressive nature of MRSA, which is notorious for its virulence and ability to cause multifocal infections, particularly in individuals with predisposing risk factors [11]. In many instances, suspicion of this fatal disease can be made based on the patient’s initial presentation and physical exam findings; however, more common pathologies should be ruled out first. CT and MRI findings remain pivotal in establishing the diagnosis. However, until imaging confirms the diagnosis, suspicion of this disease should remain low on the differential.

Once HSEA and SSA are confirmed, a multidisciplinary care team is crucial. Infectious disease specialists provide tailored guidance on antibiotic selection and duration, while neurosurgery can help evaluate the feasibility of surgical intervention. Critical care teams managed the patient’s respiratory compromise and agitation, ensuring airway protection and hemodynamic stability during the acute phase of illness. This coordinated approach was instrumental in achieving a favorable outcome, as evidenced by the patient’s eventual extubation and discharge to rehabilitation.

Consequently, since this disease is fairly rare, proper management can vary; nevertheless, current recommendations suggest a combination of surgical drainage of the abscess to ensure proper source control and antibiotic therapy, which are the gold standard for both HSEA and SSA [4-7,12]. However, this method of management has seen success based on the limited number of cases reported, and surgical intervention may not be feasible. In this unique case, there was diffuse and extensive involvement of the patient’s spinal cord (Figures 1a-2b), which posed significant challenges to surgical intervention. Therefore, due to the complex nature of these abscesses, the patient required a higher level of care, which was not available at our institution.

Subsequently, this is a unique case where the patient was able to recover from antibiotic therapy. The quick and prompt use of vancomycin, cefepime, ampicillin, and dexamethasone, due to the high clinical indication of bacterial meningitis, was a crucial moment in decreasing this patient’s mortality [13]. Once MRSA was identified, the regimen was refined to include daptomycin and ceftaroline for six weeks [11]. Fortunately, the patient responded very well to this regimen underscoring the potential for conservative management in this situation, especially when surgical intervention is not feasible. What is also key is that one prior case had recorded non-surgical interventions to manage anterior epidural abscess [14]. Therefore, considerations to use only antibiotic therapy when surgical intervention is unavailable may be considered.

A key limitation in this case was the inability to pursue surgical intervention due to the inability of our facility to provide a proper higher level of care. It should be noted that surgical management with IV antibiotics should still be the mainstay of the treatment since more studies have shown the efficacy of such treatment in this rare disease. However, this case does highlight the option to provide medical management without surgical intervention if necessary.

## Conclusions

Overall, further research is needed to establish evidence-based guidelines for the management of such a rare disease. However, blood cultures, lumbar puncture, with all other blood work, should be done shortly before starting empiric broad-spectrum antibiotic therapy, especially if there is high clinical suspicion for a bacterium causing meningitis/encephalitis. Further CT/MRI imaging can further diagnose the condition of HSEA or SSA; however, the first-line treatment should be surgical intervention with IV antibiotics based on prior successful studies. However, this case shows that, if surgical intervention is not feasible, IV antibiotics can be considered for six weeks, starting with more broad-spectrum and eventually more targeted therapy based on blood cultures.
